# Nedosiran Safety and Efficacy in PH1: Interim Analysis of PHYOX3

**DOI:** 10.1016/j.ekir.2024.02.1439

**Published:** 2024-03-04

**Authors:** Jaap Groothoff, Anne-Laure Sellier-Leclerc, Lisa Deesker, Justine Bacchetta, Gesa Schalk, Burkhard Tönshoff, Graham Lipkin, Sandrine Lemoine, Thomas Bowman, Jing Zhou, Bernd Hoppe

**Affiliations:** 1 Department of Pediatric Nephrology, Emma Children’s Hospital, Amsterdam UMC, University of Amsterdam, Amsterdam, The Netherlands; 2 Pediatric Nephrology Rheumatology Dermatology Unit, Reference Center for Rare Renal Diseases, ORKID and ERK-Net networks, Lyon University Hospital, Bron, France; 3 Pediatric Nephrology Center Bonn, Bonn, Germany; 4 Department of Pediatrics, University Children’s Hospital, Heidelberg, Germany; 5 Department of Nephrology, University Hospitals Birmingham, Birmingham, UK; 6 Department of Nephrology, Reference Center for Rare Renal Diseases, ORKID, University of Lyon, Lyon, France; 7 Dicerna Pharmaceuticals, Inc., a Novo Nordisk Company, Lexington, Massachusetts, USA; 8 German Hyperoxaluria Center, Pediatric Nephrology Center Bonn, Bonn, Germany

**Keywords:** glyoxylate metabolism, nedosiran, primary hyperoxaluria type 1, RNAi therapy, safety/efficacy, urinary oxalate excretion

## Abstract

**Introduction:**

Primary hyperoxaluria (PH) is a rare genetic disorder of hepatic glyoxylate metabolism. Nedosiran is an RNA interference (RNAi) therapeutic that the US Food and Drug Administration has approved for treatment of PH1. PHYOX3 is a trial evaluating monthly nedosiran in patients with PH.

**Methods:**

In this PHYOX3 interim analysis, participants with PH1 who continued from a single-dose nedosiran trial (PHYOX1), with no previous kidney or liver transplantation, dialysis, or evidence of systemic oxalosis were eligible. The safety and efficacy of once-monthly nedosiran was assessed over 30 months.

**Results:**

Thirteen participants completed PHYOX1 and continued into PHYOX3. At baseline, the mean (SD) and median (range) age was 24.2 (6.6) years and 23.0 (14–39) years, respectively; 53.8% were female and 61.5% were White. Mean estimated glomerular filtration rate (eGFR) remained stable (62–84.2 mL/min per 1.73 m^2^) to month 30. Mean 24-hour urinary oxalate (Uox) excretion showed a sustained reduction from baseline of ≥60% at every visit (months 2–30). From month 2, at least 10 of 13 (76.9%) participants achieved normal (<0.46 mmol/24h; upper limit of assay-normal [ULN]) or near-normal (≥0.46 to <0.60 mmol/24h; ≥ULN to <1.3 × ULN) 24-hour Uox excretion. All participants experienced ≥1 adverse event (AE), mostly mild or moderate in severity (primarily, injection site events). Three serious, not treatment-related AEs were reported; there were no deaths or study discontinuations due to AEs.

**Conclusion:**

Nedosiran was well-tolerated in patients with PH1, and treatment resulted in a sustained, substantial reduction in Uox excretion for at least 30 months in this long-term study. No safety signals have been identified to date. The PHYOX3 study is ongoing.

## Graphical abstract

**Figure:**
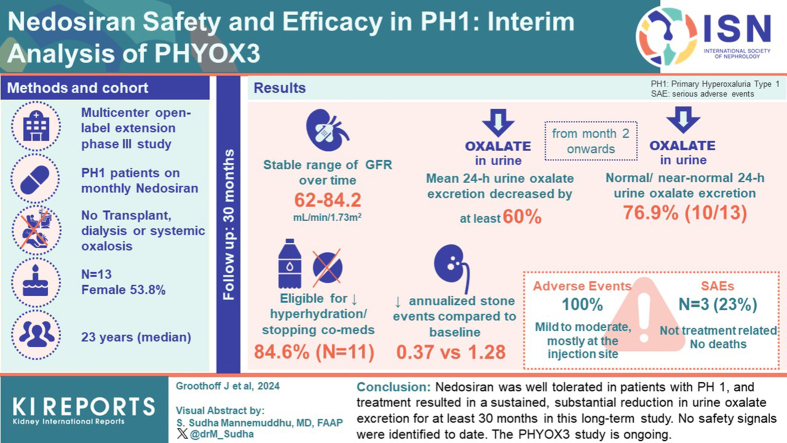

The PHs are a family of rare, autosomal recessive genetic disorders of hepatic glyoxylate metabolism.^1^, ^2^, ^3^, ^4^, ^5^ Each of the 3 genetically different subtypes of PH (PH1, PH2, and PH3) have a distinct enzyme deficiency that leads to endogenous oxalate overproduction by conversion of glyoxylate via hepatic lactate dehydrogenase.^1^^,^^4^^,^^6^, ^7^, ^8^, ^9^ The excess oxalate, which is a metabolic end product in humans, is mostly excreted by the kidneys, thus inducing hyperoxaluria. The elevated Uox excretion often leads to precipitation and deposition of calcium oxalate crystals in the renal tubules and kidney parenchyma. This ultimately leads to the formation of kidney stones and/or nephrocalcinosis, and subsequently, progressive kidney damage and chronic kidney disease (CKD). If left untreated, severe CKD will eventually impair oxalate excretion to an extent that it causes systemic storage of oxalate in various tissues (such as skin, eyes, bones, and heart), causing life-threatening and disabling multiorgan disease called systemic oxalosis.^1^^,^^4^^,^^5^^,^^10^, ^11^, ^12^, ^13^, ^14^, ^15^, ^16^, ^17^, ^18^, ^19^, ^20^

Nedosiran is an RNAi therapeutic approved by the US Food and Drug Administration for the treatment of patients with PH1 aged ≥9 years with an eGFR ≥30 mL/min per 1.73 m^2^ and is under investigation for the treatment of PH2 and PH3.^21^, ^22^, ^23^, ^24^ Nedosiran impedes the final step of the oxalate overproduction in the glyoxylate pathway by inhibiting hepatic lactate dehydrogenase production (encoded by hepatic *LDHA*). Due to its mechanism of action, nedosiran has potential utility in patients with all 3 known genetic subtypes of PH.^7^^,^^22^, ^23^, ^24^, ^25^, ^26^ In the single-dose, open-label, PHYOX1 study of participants with PH1 or PH2, subcutaneous administration of nedosiran (1.5, 3.0, and 6.0 mg/kg) was generally well-tolerated, had a favorable safety profile, and induced a 42.6% reduction in 24-hour Uox excretion from baseline to day 57.^22^ In the double-blind, placebo-controlled, PHYOX2 study, once monthly nedosiran administered at a weight-based dosage of 3.5 mg/kg in children with PH1 (not exceeding 136 mg), and fixed dosages of 136 mg and 170 mg in PH1 individuals weighing <50 kg and ≥50 kg, respectively, was generally well-tolerated and induced a significant 59% mean placebo-adjusted reduction in 24-hour Uox excretion from baseline between day 90 and day 180.^23^

Given that patients with different PH types have different clinical characteristics, the ongoing PHYOX3 study was designed to evaluate the long-term safety and efficacy of monthly nedosiran administration in treating patients with PH1, PH2, and PH3. Herein, we report interim safety and efficacy data on 13 participants with PH1 who completed PHYOX1 and continued on in PHYOX3. Results from patients with PH2 and PH3 will be reported in future publications (see Supplementary Material).

## Methods

### Study Design and Participants

PHYOX3 (NCT04042402) is a multicenter, uncontrolled, open-label extension phase 3 trial evaluating long-term safety and efficacy of monthly nedosiran in patients with genetically confirmed PH1, PH2, and PH3 who completed a previous trial of nedosiran, and PH-affected pediatric siblings of previous nedosiran clinical trial completers. Participants were recruited from July 2019 to March 2020, then underwent treatment and were followed-up with until November 2022 (the cutoff for this publication). The washout period between PHYOX1 and PHYOX3 ranged from 10 to 23 months. The full methodology and results of the parent studies have been published previously.^22^, ^23^, ^24^

**Figure 1:**
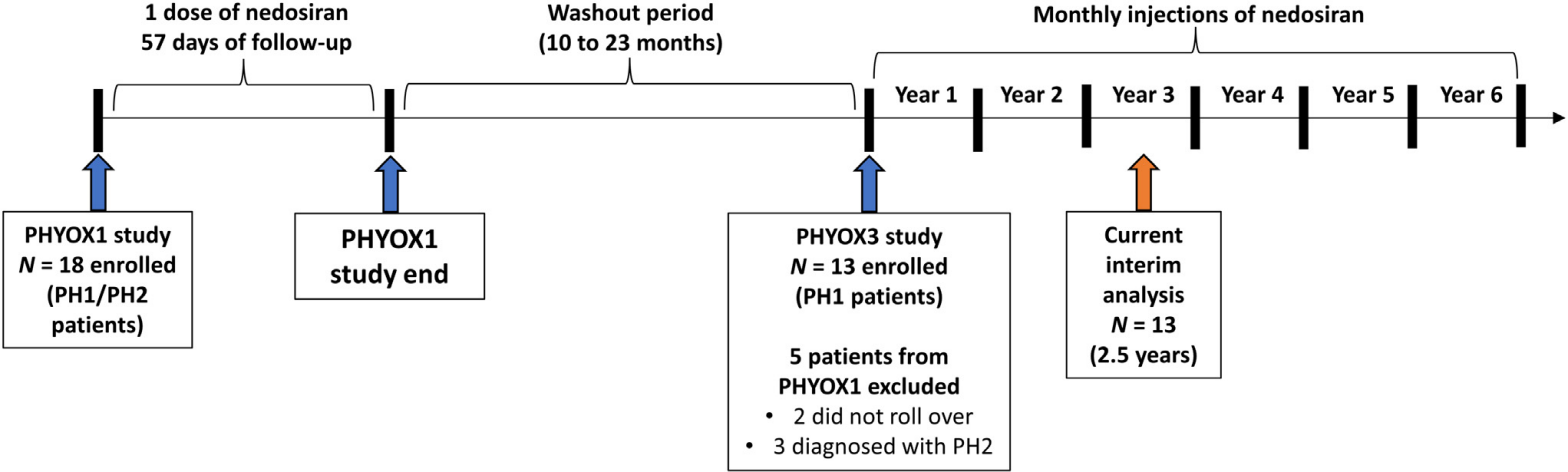
Study design. PH, primary hyperoxaluria. Participant flow for the PHYOX1 study rollover, washout period, and PHYOX3 study.

This interim analysis of PHYOX3 evaluated PHYOX1 PH1 completers (Figure 1), who were aged ≥12 years, had baseline 24-hour Uox excretion ≥0.7 mmol (63 mg) for patients aged ≥18 years (or ≥0.7 mmol per 1.73 m^2^ body surface area [63 mg/1.73 m^2^] for patients aged <18 years), and had an eGFR ≥30 mL/min per 1.73 m^2^ at baseline in the PHYOX3 study.^22^ For entry into PHYOX3, eGFR at screening was calculated using the CKD-Epidemiology Collaboration equation in adults, or the multivariate 2012 formula by Schwartz in patients aged 6 to <18 years.^27^^,^^28^ Key exclusion criteria included the following: previous renal or hepatic transplantation or planned transplantation during study period, current dialysis, plasma oxalate level >30 μmol/l (the threshold for plasma calcium oxalate supersaturation), documented evidence of clinical manifestations of systemic oxalosis, previous use of an RNAi drug other than nedosiran in the past 6 months, or history of reactions to oligonucleotide-based therapy (i.e., severe thrombocytopenia, hepatotoxicity, severe flu-like symptoms, localized skin reactions, and coagulopathy or clinically significant prolongation of clotting time). Participants receiving oral pyridoxine (vitamin B6) must have been at a stable dose for ≥4 weeks prior to day 1 and be willing to remain on the same stable dose throughout the study.

PHYOX3 was conducted in compliance with the Declaration of Helsinki, the International Conference on Harmonization Guidelines for Good Clinical Practice, and applicable national and local regulatory requirements. The study protocol was approved by the independent ethics committees or institutional review boards at each participating site. All enrollees provided written informed consent before participation.

### Interventions

All eligible participants received a single dose of nedosiran in PHYOX1 before enrollment into PHYOX3 with a washout period between the PHYOX1 dose and PHYOX3 enrollment. To be eligible for PHYOX3, patients in PHYOX1 initially had to show 24-hour Uox excretion values ≥80% of the lowest predose screening value. This criterion was intended to account for intraperson variability in 24-hour Uox excretion when assessing the duration of pharmacodynamic effect after administration of single-dose nedosiran. In PHYOX3, patients then received a fixed, monthly dose of nedosiran. The nedosiran dosage was based on weight as follows: adults and adolescents aged 12 to 17 years with weight ≥50 kg received 160 mg (1 mL volume, free acid; equivalent to 170 mg sodium salt); adults and adolescents weighing <50 kg received 128 mg (0.8 mL volume, free acid; equivalent to 136 mg sodium salt); and children aged 6 to 11 years (inclusive) were to receive 3.3 mg/kg (3.5 mg/kg sodium salt) not to exceed 128 mg.

Nedosiran was administered by study staff via subcutaneous injection using 25-gauge to 27-gauge needles at clinic visits up to day 180, when this task could then be performed by the participant (or caregiver) under the supervision of the study staff, to confirm proper technique. At-home administration of nedosiran (optional) by participants and/or their caregivers was allowed after day 180 for those months at which a clinic visit was not required. Home health aides were available to assist participants as necessary. All participants were asked to continue their standard-of-care measures for PH (especially for vitamin B6 use). Because vitamin C, and diet, can affect Uox excretion in patients with PH,^29^ all participants were instructed to avoid vitamin C supplements, including multivitamins, and to avoid oxalate-rich foods at all times during the study. The planned duration of treatment in PHYOX3 is up to 6 years, or until nedosiran is commercially or otherwise available to a participant.

### Outcomes

The primary objective of PHYOX3 is to evaluate the effect of nedosiran on eGFR in participants with PH, measured by the annual rate of decline in eGFR. Because annual rate of decline in eGFR data were too preliminary to analyze at the time of this interim analysis, only descriptive summaries of eGFR at each visit and percent change from baseline are presented here. One of the key secondary objectives was evaluation of nedosiran safety and tolerability. This was measured by AE monitoring, change from baseline in 12-lead electrocardiogram, physical examination findings, vital signs, and clinical laboratory tests (hematology, chemistry, coagulation parameters, and urinalysis). Participants were monitored for certain potential risks related to RNAi molecules, including injection site reactions, liver abnormalities, markers of inflammation (cytokine release), and direct (antidrug antibodies) and/or indirect (antidouble-stranded DNA antibodies) markers of antibody formation against nedosiran. AEs of special interest were defined as noteworthy events for the product or product class that the sponsor intended to monitor carefully. Here, AEs of special interest included injection site reactions, muscle pain or weakness, and kidney stone events. Patients were evaluated for any new AEs and the status of existing AEs at each study visit, including proactive questioning on the occurrence of muscle pain or weakness. AEs were also reported by the participant or caregiver as either unsolicited reports or in response to general questioning. AEs were coded using the Medical Dictionary for Regulatory Activities (version 26.0) and graded according to intensity categories.

Secondary efficacy end points pertaining to Uox burden included the proportion of participants attaining normal (<0.46 mmol/24h; ULN)^30^ or near-normal (≥0.46 to <0.60 mmol/24h; ≥ULN to <1.3 × ULN)^22^ Uox excretion (adjusted per 1.73 m^2^ body surface area in all participants) at each time point throughout the study, absolute and percent maximum reduction in 24-hour Uox excretion from baseline, and percentage of participants with spot Uox-to-creatinine ratio ≤ULN or 1.5 × ULN at each time point. The limit of 1.3 × ULN for 24-hour Uox and 1.5 × ULN for spot Uox-to-creatinine ratio are historical limits set for the PHYOX clinical program. In addition to 24-hour urine collections, spot urine samples were collected to enable further analysis of the relationship between 24-hour Uox and spot Uox assessments (protocol amendment 5). Additional secondary objectives included the following: clinically apparent stone events and stone burden over 12-month periods assessed via history of stone events and baseline kidney ultrasounds (and nephrocalcinosis grading), plasma oxalate levels (for safety laboratory assessment), incidence of severe CKD (defined as CKD stage 4, eGFR = 15–29 mL/min, or stage 5 kidney failure, eGFR <15 mL/min [adjusted for body surface area in participants <18 years of age]), and proportion of participants eligible for a reduction in their original hyperhydration regimens or discontinuation of other comedications that prevent calcium oxalate formation due to attainment of normal 24-hour Uox excretion at 3 consecutive time points.

Urine collections were preservative-free and acidified (pH <2) at collection to ensure calcium oxalate solubility. Uox and plasma oxalate levels were determined centrally using an enzymatic oxidation assay catalyzed by oxalate oxidase followed by measurement of hydrogen peroxide produced in a peroxidase-catalyzed reaction. The central laboratory ULN for plasma oxalate was <10 μmol/l.

To calculate the baseline kidney stone event rate, participants in PHYOX3 were asked to provide an estimate of the number of stone events they had in the 12 months before the baseline. For the event rate during the study (i.e., after first dose in the PHYOX3 study), the number of stone events was determined using clinical data (including ultrasound). To provide a more reliable estimate of the baseline stone event rate for comparison, data on stone events in 11 patients with PH1 who were assigned to the placebo group of the PHYOX2 study (NCT03847909) were analyzed (unpublished data). A fair comparison between the 2 cohorts was possible because the stone event rate in PHYOX2 was calculated in the same manner as that of the posttreatment period in PHYOX3.

### Analysis Populations

The safety population included all participants who received ≥1 dose of nedosiran. All safety and efficacy analyses were conducted in the safety population.

### Statistics

This interim analysis includes data up to the cut-off date of November 2022. This data cutoff occurred 2.5 years into the PHYOX3 study. Summary statistics for continuous variables included mean, SD, SEM, and median (minimum, maximum). Categorical variables are presented as number and percentage of participants (*n* [%]). All analyses were performed using SAS (version 9.4; SAS Institute Inc., Cary, NC). Participants with missing data were excluded from the summary of the variable with missing data.

## Results

### Participants

Of the 15 participants with PH1 who received a single dose of nedosiran in PHYOX1, 13 enrolled in PHYOX3 across 4 sites in the Netherlands, France, Germany, and the UK. Two patients did not enroll because they received lumasiran (OXLUMO; Alnylam Pharmaceuticals, Cambridge, MA) near the start of PHYOX3. At the interim cut-off date, all 13 participants had attended their PHYOX3 month 30 visit, were still on treatment, and none had withdrawn.

At PHYOX3 baseline, the mean (SD) and median (range) age of the overall population was 24.2 (6.64) years and 23.0 (14–39) years, respectively; 53.8% of participants were female, and 61.5% were White. Two participants (15.4%) were in the 12 to <18 years age range (aged 14 and 16 years), and 11 (84.6%) were ≥18 years of age; there were no children aged 6 to 11 years in this interim analysis. Mean (SD) 24-hour Uox excretion was lower at baseline in PHYOX3 than in PHYOX1: 0.88 (0.27) mmol/24h per 1.73 m^2^ and 1.34 (0.54) mmol/24h per 1.73 m^2^, respectively, because patients were previously treated with a single-dose of nedosiran in PHYOX1. Mean (SD) baseline eGFR was 77.6 (21.82) mL/min per 1.73 m^2^ (corresponding to CKD stage 2). At PHYOX3 baseline, most participants (11 [84.6%]) had eGFRs in the ≥45 mL/min per 1.73 m^2^ category (*n* = 2 for ≥30 and <45 mL/min per 1.73 m^2^). The majority of participants (8 of 13, 61.5%) were in CKD stage 1 or 2. Two participants (15.4%) reported kidney stone events in the 12 months prior to baseline. The mean (SD) number of kidney stones at baseline (based on data from 11 participants [84.6%]) was 10.1 (23.98). Vitamin B6 use was reported by 7 participants (53.8%) at baseline (Table 1).

**Table 1:** Demographics and disease characteristics at PHYOX3 baseline

Parameter	Participants with PH1 (*N* = 13)
Age, yr	
Mean (SD)	24.2 (6.64)
Median (range)	23.0 (14–39)
Age group, *n* (%)	
<6 years	0
6–11 years	0
12–17 years	2 (15.4)
≥18 years	11 (84.6)
Sex,^a^*n* (%)	
Female	7 (53.8)
Male	6 (46.2)
Race, *n* (%)	
White	8 (61.5)
Asian	1 (7.7)
Not available	4 (30.8)
Ethnicity	
Not Hispanic or Latino	9 (69.2)
Not available	4 (30.8)
eGFR, mL/min per 1.73 m^2^	*n* = 13
Mean (SD)	75.5 (22.2)
Median (range)	78.0 (36–114)
eGFR Category	
≥30 and <45 mL/min per 1.73 m^2^	2 (15.4)
≥45 mL/min per 1.73 m^2^	11 (84.6)
PHYOX1 baseline 24-hour Uox excretion^b^^,^^c^	
Mean (SD), mmol/24h per 1.73 m^2^	1.34 (0.54)
Median (range), mmol/24h per 1.73 m^2^	1.12 (0.76–2.23)
Mean, mg/24h per 1.73 m^2^	120.6
PHYOX3 baseline 24-hour Uox excretion^b^^,^^c^	
Mean (SD), mmol/24h per 1.73 m^2^	0.88 (0.27)
Median (range), mmol/24h per 1.73 m^2^	0.93 (0.53–1.39)
Mean, mg/24h per 1.73 m^2^	79.2
Time between dosing in PHYOX1 and baseline in PHYOX3, months	10–23
Vitamin B6 use, yes/no	7 (53.8) / 6 (46.2)
Any kidney stone event in past 12 months (patient reported), yes/no	2 (15.4) / 11 (84.6)
Number of kidney stone events in past 12 months^d^	*n* = 2
Mean (SD)	1.0 (0)
Median (range)	1.0 (1–1)
Number of kidney stones at baseline	*n* = 11
Mean (SD)	10.1 (23.98)
Median (range)	2.0 (0–81)
Surface area of kidney stones at baseline, mm^2^	*n* = 8
Mean (SD)	78.5 (92.03)
Median (range)	43.0 (0–250)
Time since PH diagnosis, years	
Mean (SD)	15.753 (5.4119)
Median (range)	15.55 (4.12–25.07)
Chronic kidney disease stage^e^	
Stage 1	3 (23.1)
Stage 2	5 (38.5)
Stage 3A	0
Stage 3B	3 (23.1)
Stage 4	0
Stage 5	0
Missing	2 (15.4)

eGFR; estimated glomerular filtration rate; PH, primary hyperoxaluria.

a Biological sex at birth.

b Body surface area (BSA) adjusted 24-hour Uox excretion was used for all participants.

c To convert mmol/24h per 1.73 m^2^ to mg/24h per 1.73 m^2^, mmol values are multiplied by 90.

d The summary only applies to patients who answered “yes” to having a kidney stone event in the past 12 months.

e Based on medical history during patient enrollment rather than baseline eGFR value.

### Efficacy

**Figure 2:**
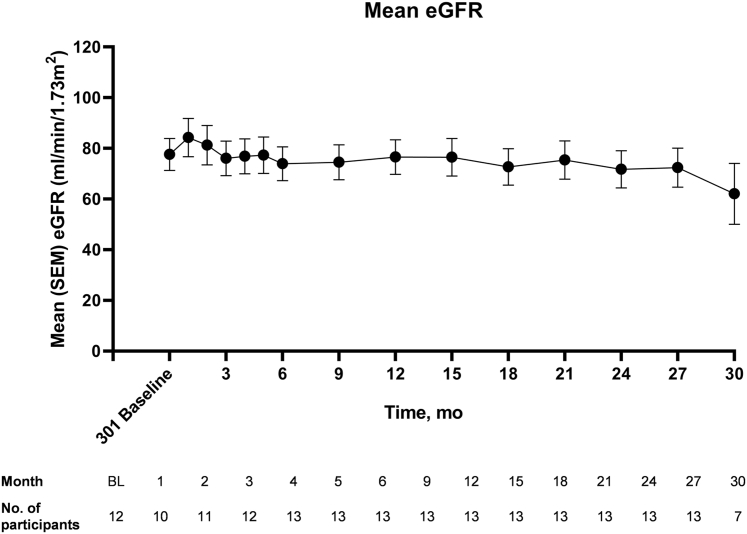
Mean eGFR throughout the study. eGFR, estimated glomerular filtration rate. Mean (± SEM) eGFR at PHYOX3 baseline (“301 baseline”) and throughout the study. Number of participants assessed at each time point is shown below the graph.

**Figure 3:**
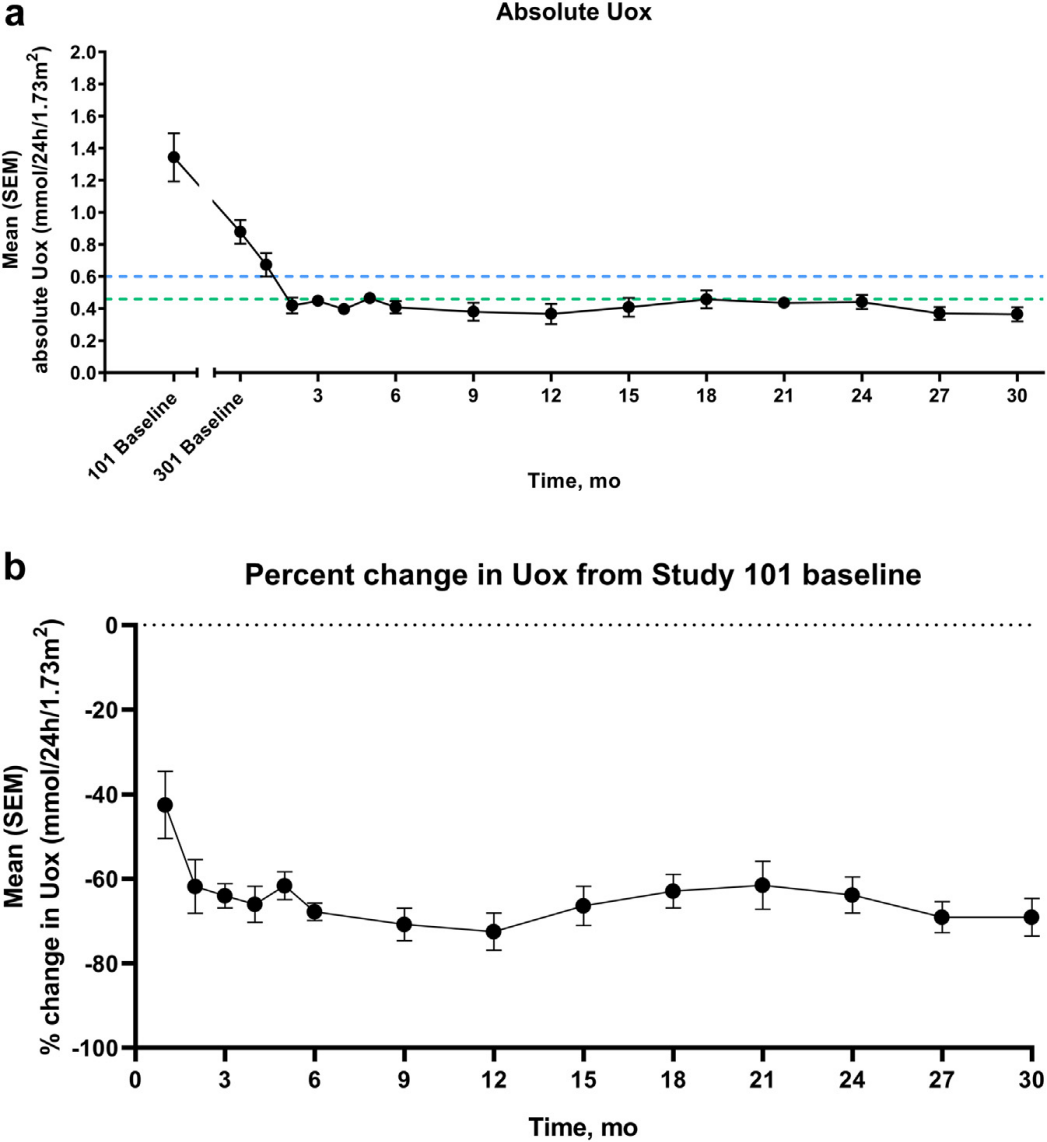
Mean absolute change, percent change, and maximum percent reduction in 24-hour Uox from baseline. (a) Mean (± SEM) absolute urinary oxalate (Uox) at PHYOX1 baseline (“101 baseline”), PHYOX3 baseline (“301 baseline”), and throughout the study. The number of participants assessed at each time point was 13 for all months except month 2 (*n* = 12). Dotted lines indicate normal (green) and near-normal (blue) limits. (b) Mean (SEM) percent change in Uox throughout the study. The number of participants assessed at each time point is shown below the graph. SEM, standard error of the mean; Uox, urinary oxalate.

**Figure 4:**
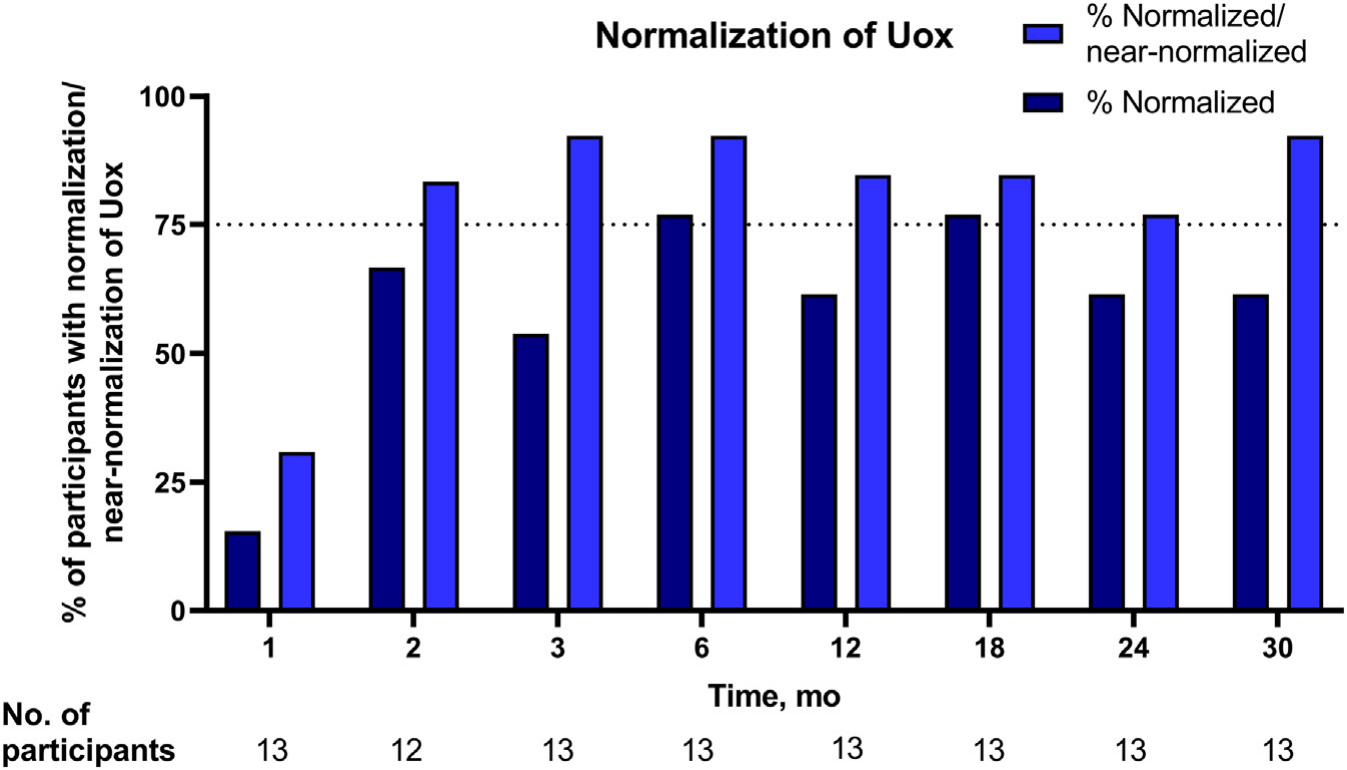
Percentage of participants with normalization or normalization/near-normalization of Uox excretion throughout the study. The number of participants assessed at each time point is shown below the graph. Normalization is defined as <0.46 mmol/24h (ULN); near-normalization is defined as ≥0.46 to <0.60 mmol/24 hours (≥ULN to <1.3 × ULN). ULN, upper limit of normal; Uox, urinary oxalate.

Mean eGFR remained stable in this cohort throughout the study, up to month 30 (Figure 2). During the single-dose PHYOX1 study, mean (SD) 24-hour Uox excretion levels fell by 42.6% (32.4) from baseline to day 57. From day 57 in PHYOX1 to PHYOX3 baseline (representing a washout period of 10–23 months), mean (SD) Uox excretion rose from 0.72 (0.40) to 0.88 (0.27) mmol/24h per 1.73 m^2^. After retreatment with monthly nedosiran in PHYOX3, 24-hour Uox excretion again fell, and excretion was sustained at normal or near-normal levels throughout the study period starting from month 2 (Figure 3a). At each study visit in PHYOX3, a mean percent reduction in 24-hour Uox excretion of at least 60% from PHYOX1 baseline was maintained from months 2 to 30 (Figure 3b). Similarly, starting at month 2, more than 80% of participants had spot Uox-to-creatinine ratios ≤1.5 × ULN at each visit. At each study visit starting at month 2, at least 10 (>75%) participants achieved normal or near-normal 24-hour Uox excretion (Figure 4).

A total of 11 participants (84.6%) were eligible for reduction of hyperhydration and discontinuation of other comedications during the study at any visit. To be eligible for hyperhydration reduction or comedication discontinuation, a participant needed to achieve normalization of Uox excretion on 3 consecutive visits.

The baseline annualized stone event rate was 0.1538 (2 participants [15%], 2 events, 13 years exposed) based on recall data provided by participants. The annualized stone event rate based on posttreatment clinical data collected during the study period was 0.37 (5 participants [38.5%], 12 events, 32.37 years exposed). By comparison, the PHYOX2 placebo group (*n* = 11, mean [SD] baseline 24-hour Uox: 1.96 [0.71] mmol/d)^23^ had an annualized event rate during the study of 1.28 (4 participants [36.4%], 7 events, 5.49 years exposed) (Table 2). All kidney stone events observed in PHYOX3 were considered mild or moderate in severity and had recovered or resolved by the data cutoff date.

**Table 2:** Kidney stone events

Variable, *n* (%)	Participants with PH1 in PHYOX3 (*N* = 13)	Participants with PH1 in PHYOX2 Placebo group (*N* = 11)
Kidney stone events during study period^a^		
Participants, *n* (%)^b^	5 (38.5)	4 (36.4)
Events, *n*	12	7
Total years exposed	32.37	5.49
Annualized stone event rate	0.37	1.28

PH, primary hyperoxaluria.

a Number of years exposed during study period is the total elapsed time between the first dose date and last day of visit in the study, divided by 365.25. Annualized stone event rate (number of stone events/yr/person) is derived as number of stone events in the exposure period ÷ total number of years exposed. The number of stone events is calculated as the number of discrete (nonconcurrent) adverse events marked as a kidney stone event on the adverse event case report form. Kidney stone events include renal stone requiring medical intervention, stone passage with or without hematuria, and renal colic requiring medication. Concurrent events are defined as events occurring within the same 4-week (28-day) window.

b The count of the number of nonconcurrent stone events may include multiple events per subject.

### Safety

All participants experienced at least 1 treatment-emergent AE (TEAE). Most TEAEs reported were mild or moderate in severity; 5 participants (38.5%) reported mild AEs, and 7 (53.8%) reported moderate AEs. Three participants (23.1%) reported serious AEs as follows: 1 participant with urolithiasis, obstructive kidney stone, acute kidney injury, and pain in left arm; 1 participant with pyelonephritis associated with a preexisting kidney stone; and 1 participant with kidney failure, which was also considered a severe AE. These SAEs were deemed not related to nedosiran. There were no deaths or AEs leading to discontinuation of the study drug to date (2.5-year cutoff).

Ten of 13 participants (76.9%) had treatment-related TEAEs; injection site AEs were the most common (82.7%) treatment-related TEAEs. Eight participants (61.5%) reported AEs of special interest, including injection site reactions (3 [23.1%]), kidney stone events (5 [38.5%]), and muscle pain or weakness (1 [7.7%]) (Table 3). Of 398 total injections in the study, 2.5% of injections had an injection site reaction. All AEs of special interest resolved during the study. No clinically relevant trends were noted for laboratory assessments.

**Table 3:** Safety summary

Variable, *n* (%)	Participants with PH1 in PHYOX3 (*N* = 13)
Any TEAE	13 (100)
Treatment-related	10 (76.9)
AEs leading to discontinuation	0
Serious AEs	3 (23.1)
Serious and treatment-related	0
Severity	
Mild	5 (38.5)
Moderate	7 (53.8)
Severe	1 (7.7)
Deaths	0
AESIs, subject *n* (%)/event *n*	
Kidney stone events^a^	5 (38.5) / 15
Injection site reactions	3 (23.1) / 10
Muscle pain or weakness	1 (7.7) / 12

AE, adverse event; AESI, adverse event of special interest; PH, primary hyperoxaluria; TEAE, treatment-emergent adverse event.

a Combined total of renal stone requiring medical intervention, stone passage with or without hematuria, and renal colic requiring medication.

## Discussion

These interim findings of the PHYOX3 study show stable kidney function among 13 patients with PH1 who received monthly nedosiran administration over 2.5 years. The safety profile of nedosiran in PHYOX3 was consistent with that observed for nedosiran in other clinical trials.^22^^,^^23^ TEAEs were generally mild-to-moderate, with injection site events being the most common treatment-related TEAE. No new safety concerns were identified. Nedosiran treatment was associated with durable and robust mean reductions in 24-hour Uox excretion, where Uox excretion was sustained in the normal or near-normal range at month 2 through month 30, in more than 75% of recipients; and stone burden appeared to be reduced.

These efficacy results are reproducible because Uox excretion was increased after the end of PHYOX1 (during the washout period), and then reduced again once treatment resumed during PHYOX3. This observation is unlikely due to concomitant vitamin B6 therapy, which was received by 53.8% of PHYOX3 participants. In PHYOX2, a similar proportion of PH1 participants in the nedosiran and placebo arms also used vitamin B6.^23^ A *post hoc* analysis showed that a significant difference in the area under the curve^23^ of percent reduction from baseline in 24-hour Uox excretion between days 90 and 180 was detected in the nedosiran arm, versus placebo arm, among those who received vitamin B6 at baseline (least squares mean [SE] of the difference between treatment group and placebo group: 5777.5 [1526.6], 95% confidence interval: 2785.4–8769.7, *P* < 0.0001) compared with those who did not receive vitamin B6 at baseline (least squares mean [SE] difference between the treatment groups: 4516.9 [2172.6], 95% confidence interval: 257.9–8776.0, *P* = 0.019). Such a *post hoc* subgroup analysis is not possible in PHYOX3 because although patients were asked to continue their standard-of-care regimen (including vitamin B6 use), their level of adherence to medications was not specifically collected; in addition, the population size is small.

Bearing in mind the small sample sizes, stone events in PHYOX3 were numerically lower than those observed among PH1 participants in the placebo group of PHYOX2 (annualized event rate, 0.37 vs. 1.28). This comparison was calculated using clinical data during the PHYOX3 and PHYOX2 on-treatment periods, and as such, is a more informative comparison than using the PHYOX3 baseline event rate, which is estimated from patient recall. In 2 other studies of patients with PH1 who were RNAi treatment-naive, 85% of participants^31^ or 95% of participants^32^ reported a history of kidney stone events at baseline; the annualized event rate was 3.19 per person-year over the 12 months prior to RNAi treatment.^31^ Like preservation of kidney function, the reduction of kidney stone events reflects the clinical importance of nedosiran for patients with PH1 in reducing the burden of disease and helping to improve their quality of life.^33^ Given that patients with PH1 often have stone disease at the onset of therapy,^23^^,^^31^ it is not expected that patients (even those with normalized Uox) may be completely free of stone events for at least the first 6 months after starting RNAi therapy.

Hyperhydration regimens are intended to reduce kidney stone events but are extremely difficult to adhere to by patients with PH1^32^; both time consuming and difficult to maintain, especially for children. Patients with PH report that hyperhydration is one of the most burdensome aspects of PH treatment and declines in quality-of-life scores were attributed in part to the difficulty of maintaining hyperhydration.^32^ Similarly, citrate medications and other treatment options represent a quality-of-life burden for patients with PH.^30^ In the current study, 11 of the 13 participants were eligible for reduction of hyperhydration regimens due to satisfactory Uox control at normal levels. Investigator experiences during the study showed that some patients stopped or reduced alkaline citrate or hydration, whereas others had no change in comedications or hyperhydration. These results indicate that nedosiran treatment could provide meaningful improvement in patients’ quality of life.

The updated 2023 European Rare Kidney Disease Reference Network and OxalEurope consensus clinical recommendations addressed use of RNAi therapies (such as lumasiran and nedosiran) in patients with PH.^30^ The expert panel strongly recommended RNAi therapy as a treatment when PH1 is genetically established, the patient is not responding to vitamin B6 (or has a variant that is not responsive to vitamin B6), has Uox excretion >1.5 ULN, and has active stone disease, nephrocalcinosis, or renal impairment.^30^ The patients with PH1 enrolled in PHYOX3 experienced baseline mean Uox excretion of 0.88 mmol/24h per 1.73 m^2^ (i.e., >1.5 × ULN), kidney stones at baseline, and other baseline indicators that suggested RNAi treatment was warranted. The guideline panel also strongly recommended that the benefits of RNAi therapy be weighed against the potential long-term risks.

Lumasiran (OXLUMO; Alnylam Pharmaceuticals) is an RNAi therapy approved by the US Food and Drug Administration and European Medicines Agency for treatment of PH1 in pediatric and adult patients.^34^ In a phase 3 trial of lumasiran (ILLUMINATE-A) with 6-month,^31^ 12-month,^35^ 24-month,^36^ and 36-month assessments,^37^ similar results were seen as those reported for nedosiran in this PHYOX3 study. Among patients that switched from placebo to lumasiran treatment (*n* = 13), a durable reduction in 24-hour Uox was observed, with a 58% mean reduction from baseline after 30 months of lumasiran treatment and 92% of patients achieving normalization or near-normalization of Uox (≤1.5 × ULN). After 30 months of nedosiran treatment in the current study, there was a mean reduction of 69% in 24-hour Uox and 92% of patients achieved normalization or near-normalization of Uox (≤1.3 × ULN). It is important to note that the Uox normalization levels were defined differently for nedosiran than for lumasiran (≤1.3 × ULN vs. ≤1.5 × ULN, respectively). The kidney stone event rate was 0.39 per person-year based on 30 months of lumasiran treatment,^37^ which is similar to the rate (0.37 per person-year) after 30 months of nedosiran treatment. Considering the lack of head-to-head clinical studies comparing nedosiran with lumasiran, specific conclusions cannot be drawn. However, nedosiran appears to be at least similar to lumasiran in terms of safety and efficacy profiles in patients with PH1.

The main limitations of this interim analysis are the small sample size, relatively short period of follow-up, and lack of urinary glycolate and citrate data. It is important to note that the small sample size is a limiting factor inherent in all rare disease studies, given the limited population available for study. The open-label design of the trial was also a limitation because there is no comparator arm. To attempt to mitigate this issue, the study data were compared with historical data where possible. For kidney stone events in particular, event rates were compared with the PHYOX2 placebo group to provide a more meaningful comparator. Although there were differences in the baseline characteristics of the PHYOX2 placebo group and the PHYOX1 rollovers (the 24-hour Uox values were higher in the PHYOX2 placebo group), these groups were comparable in terms of mean eGFR and eGFR category.

In conclusion, in patients with PH1, treatment with nedosiran reduced 24-hour Uox excretion to the normal or near-normal range and prevented progression of the disease. The reduction in Uox excretion was robust and sustained up to Month 30. In addition, quality of life could be potentially improved with the possibility of reduction in hyperhydration. No new safety signals were observed in 2.5 years of treatment with nedosiran. Longer-term follow-up data for safety and efficacy in patients with PH1 will be available in the future, because the PHYOX3 trial is ongoing with a much larger patient cohort.

## Disclosure

JG reports consulting and being speaker for Alnylam and Novo Nordisk. A-LS-L reports consulting and speaker fees from Alnylam, Alexion, and Novartis. JB reports consulting and speaker fees from Alnylam, Novo Nordisk, and Biocodex. BH reports being a consultant to Novo Nordisk and Arbor Pharmaceuticals. SL reports consulting and speaker fees from Alnylam. TB, JZ, and BH are current or former employees of Dicerna Pharmaceuticals, Inc., a Novo Nordisk company, and may hold stock or stock options. All the other authors report no conflicting interests.

## Supplementary Material

Supplementary File (PDF)

**Strobe checklist.**

## Footnotes

Supplementary File (PDF)

**Plain language summary and STROBE checklist.**
